# Surrogate endpoints and competing risk of death in cardiac arrest research

**DOI:** 10.1186/s13054-016-1345-y

**Published:** 2016-06-29

**Authors:** Victoria A. McCredie, Damon C. Scales

**Affiliations:** Department of Critical Care Medicine, Sunnybrook Health Sciences Centre, 2075 Bayview Avenue, Room D108, Toronto, ON M4N 3M5 Canada; Interdepartmental Division of Critical Care Medicine, University of Toronto, Toronto, Ontario Canada; Institute of Health Policy, Management and Evaluation, University of Toronto, Toronto, Ontario Canada

## Abstract

We urgently need new therapies to improve outcomes after cardiac arrest. Initial studies typically target surrogate endpoints, and these studies help to inform subsequent larger trials that are powered to measure more patient-orientated clinical outcomes such as survival. The competing risk of death and premature assessment of neurological prognosis pose significant challenges to measuring these surrogate endpoints after cardiac arrest.

We urgently need new therapies to improve survival with good neurological outcomes after cardiac arrest [1]. Cardiac arrest is a major global health problem, with approximately 424,000 patients experiencing an out-of-hospital cardiac arrest each year in the USA [2]. Overall, survival rates appear to have increased marginally over time, but the majority of these patients will still die before hospital discharge [3]. Initial studies of new treatments typically target surrogate endpoints rather than survival; for example, reducing persistent precipitating pathology, organ dysfunction, and markers of secondary neurological injury [4]. Interventions that can be shown to modify rates of these surrogate endpoints can then be studied in larger, adequately powered trials targeting more meaningful clinical outcomes.

In this issue of *Critical Care*, Donnino and colleagues present the results of a randomized controlled trial targeting one such surrogate endpoint, the reversal of shock after cardiac arrest [1]. Specifically, they tested whether corticosteroids—in doses similar to those used in sepsis trials—could shorten duration of vasopressor administration [5]. The trial addresses an important clinical question, and the investigators conclude that hydrocortisone does not decrease time to shock reversal in post-cardiac arrest patients. Most readers will interpret these results as evidence that the biological signal—as measured by the surrogate endpoint of shock reversal—is insufficient to justify routine use of steroids in these patients.

However, some experts may offer other potential interpretations for the apparent lack of a biological effect, including timing of medication initiation or inclusion of patients without adrenal insufficiency. Another possible explanation is that many patients died before they could achieve shock reversal, rendering this surrogate endpoint unable to discriminate between responders and non-responders. Indeed, more than two-thirds (34/50) of patients died before hospital discharge, and it is unclear how many died before shock reversal [1]. The researchers used appropriate analytical techniques to account for this competing risk, but even the most sophisticated analyses cannot detect a change in a surrogate endpoint when most patients die before they can experience it.

Using a surrogate endpoint becomes even more problematic when it does not actually sit on the causal pathway. Ideally, a surrogate endpoint should have a clear relationship as an intermediate event occurring between the exposure of interest and the more meaningful clinical outcome [6], in this case death. However, only one patient in each group died due to refractory shock. Similar to most cardiac arrest trials, the majority of deaths (68 %; 23/34) occurred after a decision to withdraw life-sustaining therapy—classified as a ‘primary neurological withdrawal of care’. Decisions to limit life support treatments after cardiac arrest are usually based on predictions of neurological prognosis, and may have little to do with ongoing need for vasopressor support [7, 8]. This further obfuscates the interpretation of shock reversal as a surrogate endpoint.

Current evidence-based guidelines now recommend delaying neurological prognostication for at least 72 h after return of spontaneous circulation owing to the inaccuracy of clinical examinations performed before this time point [9]. As a consequence, any clinical trial testing post-resuscitation interventions—including future trials testing effects on surrogate endpoints—will need to consider ways of ensuring that neurological prognostication is appropriately delayed and that decisions to withdraw life-sustaining therapy are not based solely on subjective determinations. This type of approach was successfully implemented in the Targeted Temperature Management trial, which serves as a model for future trials of post-arrest interventions [10]. To avoid the competing risk between surrogate endpoints and deaths related to estimates of poor neurological prognosis, future trials could consider restricting the time frame for measuring surrogate endpoints to earlier than 72 h. Alternatively, trialists could choose surrogate endpoints that clearly lie on the causal pathway between the exposure of interest and subsequent decisions to withdraw life-sustaining therapy, which is the usual mode of death after cardiac arrest. These approaches would introduce additional design challenges, but could improve the interpretability of surrogate endpoints in future cardiac arrest trials.
